# Efficacy of a new combination of fipronil and permethrin against *Ctenocephalides felis* flea infestation in dogs

**DOI:** 10.1186/s13071-015-0687-7

**Published:** 2015-01-29

**Authors:** Becky Fankhauser, Pascal Dumont, Lénaïg Halos, James S Hunter, Bruce Kunkle, William R Everett, Theodore S Chester, Josephus J Fourie, Mark D Soll

**Affiliations:** Merial Limited, 3239 Satellite Blvd, Duluth, GA 30096 USA; Merial S.A.S, 29 avenue Tony Garnier, Lyon, 69007 France; BerTek, Inc, 104 Wilson Bottoms Rd, Greenbrier, AR 72058 USA; ClinVet International (Pty) Ltd, PO Box 11186, Bloemfontein , 9321 Universitas South Africa

**Keywords:** *Ctenocephalides felis*, Dog, Fipronil, Permethrin, Efficacy, Water exposure, Shampoo

## Abstract

**Background:**

Five studies were conducted to evaluate the effect of a new combination of fipronil and permethrin on cat fleas, *Ctenocephalides felis*, when applied to dogs, including dogs that underwent water exposure or shampooing.

**Methods:**

In each study, 16 dogs were allocated to two groups. Each dog was infested with 100 unfed adult fleas on Days −1, 7, 14, 21 and 28. Eight dogs were treated with a new topical spot-on formulation containing 6.76% w/v fipronil + 50.48% w/v permethrinon Day 0; and eight dogs served as untreated controls. Twenty-four or 48 h after treatment or subsequent infestation, each dog was combed to remove and count live fleas. In addition, the dogs were subjected to different levels of water or shampoo exposure. In study 1, dogs were not subjected to any water exposure or shampooing; in study 2, dogs were water immersed twice during the month on Days 10 and 24; in study 3, dogs were water immersed three times on Days 10, 17 and 24; and in studies 4 and 5, dogs were shampooed once on Day 17.

**Results:**

All groups of dogs administered a single topical treatment with a combination of fipronil and permethrin had significantly (p < 0.005) lower flea counts than untreated controls 24 h and 48 h post-treatment or post-infestation, regardless of whether they underwent water exposure/shampooing or not. The reductions in *C. felis* counts were between 98.4% and 100% at all time points in all studies.

**Conclusions:**

The new topical spot-on formulation of fipronil and permethrin maintains a high level of protection of dogs against *C. felis* flea infestations even when the dogs are exposed to environmental factors that are believed to adversely affect efficacy, such as water exposure or shampooing.

## Abrégé

**Contexte:**

Cinq études ont été conduites afin d’évaluer l’efficacité chez le chien d’une nouvelle combinaison de fipronil et perméthrine vis-à-vis de la puce du chat, *Ctenocephalides felis*, y compris en conditions incluant bains et shampooings.

**Méthode:**

Dans chaque étude, 16 chiens ont été répartis en deux groupes. Chaque chien a été infesté par 100 puces adultes à jeun aux jours −1, 7, 14, 21 et 28. Huit chiens ont été traités avec la nouvelle combinaison spot-on contenant 6,76% w/v fipronil + 50,48% w/v perméthrine au jour 0; huit chiens témoins restaient non traités. 24 ou 48 heures après traitement ou infestation, chaque chien a été peigné afin de retirer et de compter les puces vivantes. De surcroît, les chiens ont été soumis à différents degrés d’exposition à l’eau ou au shampooing. Dans l’étude 1, les chiens n’ont été ni exposés à l’eau, ni shampooinés; dans l’étude 2, les chiens étaient immergés dans l'eau deux fois dans le mois, aux jours 10 et 24; dans l’étude 3, les chiens ont été immergés dans l'eau trois fois durant le mois, aux jours 10, 17 et 24 et dans les études 4 et 5, les chiens ont été shampooinés une fois au jour 17.

**Résultats:**

Tous les chiens ayant reçu un traitement topique unique de la combinaison de fipronil et perméthrine ont présenté significativement moins de puces que les chiens témoins (p < 0.005) à 24 h et 48 h post-traitement ou post-infestations, qu’ils aient été immergés/shampooinés ou non. La réduction du nombre de *C. felis* était de 98,4% à 100% à tous les points de comptage de l’étude.

**Conclusions:**

La nouvelle formulation topique de fipronil et perméthrine en spot-on maintient un haut niveau de protection des chiens contre les infestations par les puces *C. felis*, même lorsque les chiens sont exposés à des facteurs environnementaux susceptibles d’altérer l’efficacité, comme l’exposition à l’eau ou à un shampooing.

## Background

The cat flea, *Ctenocephalides felis*, is the most important ectoparasite of dogs and cats in most areas of the world [1]. Recent studies have highlighted the high rate of flea infestation in companion animals, ranging between 12% and 47% in some European countries [2-4]. In most temperate areas, large variations in abundance are seen during the year, with a lower level in winter, but increasing from spring to fall. In some countries, such as Austria, Italy, Germany and Spain, peak infestation rates of more than 70% have been reported [3]. Infestation rates may be highly variable from one year to another, but they also depend on location (rural versus urban) and whether the pet has outdoor access. Given the pathogenic and vector potential of fleas [5], coupled with their high prevalence, the effective control of fleas represents a major objective in small animal veterinary medicine.

The recent decade has seen the development of an increased number of insecticides dedicated to the control of fleas [6]. In addition to the molecules and the specificity of their modes of action, it is very important to consider the dosage form of the insecticidal products as this is also likely to impact pet owner compliance. The established program must be adapted to the profile/habits/behaviour of the pet owners and their animals. Spot-on formulations have been demonstrated to offer convenience to the pet owner and to have enhanced compliance. Nevertheless, they may be impacted by environmental factors. Exposure to water and shampooing have the highest impact on the efficacy of topical products [7,8] and dogs are most frequently confronted with both, either through rain exposure, bathing/swimming, or shampooing. It is therefore very important to assess the efficacy of any topical product under various water exposure conditions.

Fipronil and permethrin are potent ectoparasiticides targeting the nervous system of arthropods with both acaricidal and insecticidal properties [6].

Fipronil acts by binding to GABA and glutamate receptors, which inhibits the opening of the chloride ion channels and consequently leads to neuronal hyperactivity. Glutamate receptors are specific to arthropods resulting in a wide safety margin. The spectrum of activity includes insects (fleas, lice) as well as acarines (ticks, *Sarcoptes, Cheyletiella*) [6]. Permethrin acts by opening of Na + channels, inducing nerve cell membrane depolarization. The rapid action on the cerebral ganglia results in a sudden shock of the arthropods, known as the ‘knock-down’ effect. Permethrin is volatile and its presence around treated animals explains its repellent effect, as demonstrated on flying insects [6].

A new spot-on formulation of 6.76% w/v fipronil and 50.48% w/v permethrin (Frontline Tri-Act®/Frontect®) has been developed and is designed for use as a monthly topical solution for dogs.

The five studies presented here were undertaken to evaluate the efficacy of this new spot-on formulation against different strains of adult *C. felis* when applied to dogs under experimental conditions and mimicking diverse natural water exposure scenarios (no water exposure; water immersion two or three times a month, or shampooing once).

## Methods

### Animals and study design

All animals were healthy, pure or mixed-breed dogs of both sexes. The five studies were conducted under a controlled and blinded design, with dogs randomly allocated to two groups of eight dogs each (control and treated). Prior to treatment, all dogs underwent a physical examination conducted by a veterinarian to ensure that they were healthy. To detect the presence or absence of any treatment-related or unrelated health abnormality or adverse event, health observations were conducted daily from the start to end of all studies as well as at hourly intervals for 4 h immediately after treatment. All animals were managed similarly, with due regard for their well-being and in compliance with the Merial Ethics Committee and Merial Institutional Animal Care and Use Committee requirements.

The studies were designed in accordance with the *World Association for the Advancement of Veterinary Parasitology (WAAVP) guidelines for evaluating the efficacy of parasiticides for the treatment, prevention and control of flea and tick infestation on dogs and cats* [9] and were conducted in accordance with Good Clinical Practices as described in *International Cooperation on Harmonisation of Technical Requirements for Registration* of *Veterinary Medicinal Products* [10].

Studies 1, 3, and 4 were conducted in the United States and studies 2 and 5 were conducted in South Africa.

### Flea strains

Infestations were induced with three different laboratory-maintained *C. felis* strains not known to be resistant to any ectoparasiticide. Fleas used in study 1 were obtained from the Bertek, Inc. colony (Greenbrier, Arkansas), which was started in 2004 using US sourced fleas. Wild caught fleas were added to this colony in 2011. US PLRS strain fleas were used in studies 2 and 5. This strain originated from the US and had been maintained in colony in South Africa for approximately 20 months prior to being used in these studies. Fleas from Elward II labs (Soquel, California) were used in studies 3 and 4. This strain has been maintained under laboratory conditions for approximately 33 years with the addition of wild caught fleas to the colony approximately every three to six months.

### Treatment

Dogs assigned to the five control groups were not treated. On study Day 0, each dog in the five treated groups received a topical application of 6.76%w/v fipronil + 50.48% w/v permethrin, (Frontline Tri- Act®/Frontect®) at either the minimum recommended dose of 0.1 mL/kg bodyweight corresponding to a minimum dose of 6.76 mg/kg fipronil and 50.48 mg/kg permethrin (study 1) or the commercial dose of the product based on bodyweight (pipette dose, studies 2, 3, 4 and 5). For studies 2 through 5, the applied fipronil dose ranged from 6.90 mg/kg to 13.39 mg/kg and the permethrin dose ranged between 51.51 mg/kg and 99.96 mg/kg. Treatments were applied once on Day 0 directly on the skin, after parting the hair, at two spots on the midline of the neck, between the base of the skull and the shoulder blades.

### Flea infestations and adult flea counts

Each dog was infested with 100 (±5) unfed adult fleas on Days −1, 7, 14, 21 and 28. All live fleas remaining on the dogs were removed and counted via thorough combing of all body areas with a fine-tooth flea comb either on Day 1 at 24 h after treatment and at 24 h after each of the subsequent weekly flea infestations for study 1, or at 48 h after treatment or subsequent weekly infestation for studies 2, 3, 4 and 5.

### Water exposure/shampooing

No water exposure or shampooing was performed in study 1. In studies 2 and 3, dogs were submitted to water immersion carried out by thoroughly wetting dogs (including the head) with spray from a bathing wand for at least 1 min. The dogs were dried with a blow dryer before returning them to their cages (study 2) or allowed to air dry (study 3). Water immersion was conducted on Days 10 and 24 in study 2 and on Days 10, 17 and 24 in study 3. In studies 4 and 5, dogs were shampooed following a similar protocol for both studies on Day 17 with Bio Guard® shampoo (Farnam Companies, Inc., USA) as follows: the coat of the dog was wetted thoroughly with warm water. The shampoo was spread across the animal to form a foamy lather. It was massaged into the wet coat of the entire animal and left on for 5 min. The animal was then rinsed with clean water. The dogs were dried with a blow dryer before returning them to their cages (study 5) or were allowed to air dry (study 4).

### Data analysis

For the evaluation of efficacy against adult fleas, the flea counts were transformed to the natural logarithm of (count + 1) for calculation of geometric means for each treatment group as previously described and in accordance with WAAVP guidelines [9]. The percent efficacy was calculated as 100×[(C-T)/C], where C is the geometric mean of the untreated dogs and T is the geometric mean of the treated dogs. The log counts of the treated groups were compared to the log counts of the untreated groups using an F-test adjusted for the allocation blocks used to randomize the dogs to treatment group. The mixed procedure in SAS® version 9.1.3 was used for the analysis, with the treatment groups listed as a fixed effect and the allocation blocks listed as a random effect. All testing was two-sided at the significance level of p <0.05.

## Results

The adult flea counts throughout the studies in the treated and untreated groups are summarized in Table 1. The geometric means of the flea counts of untreated dogs ranged from 55.3 to 97.9.

**Table 1:** **Geometric mean adult flea (** ***Ctenocephalides felis)*** **counts and percent efficacy relative to non-treated controls for dogs treated with a topical spot-on formulation of fipronil and permethrin**

**Water exposure study days**	**No water exposure**			**2 or 3 water immersions (on D10, and 24, or on D10, D17 and 24, respectively)**						**1 shampooing (on D17)**					
	**Study 1** ^**1**^			**Study 2** ^**2**^			**Study 3** ^**2**^			**Study 4** ^**2**^			**Study 5** ^**2**^		
	**Flea counts**		**Efficacy (%)**	**Flea counts**		**Efficacy (%)**	**Flea counts**		**Efficacy (%)**	**Flea counts**		**Efficacy (%)**	**Flea counts**		**Efficacy (%)**
	**Control group** ^**3**^	**Treated group** ^**3**^		**Control group** ^**3**^	**Treated group** ^**3**^		**Control group** ^**3**^	**Treated group** ^**3**^		**Control group** ^**3**^	**Treated group** ^**3**^		**Control group** ^**3**^	**Treated group** ^**3**^	
	**(n = 8)**	**(n = 8)**		**(n = 8)**	**(n = 8)**		**(n = 8)**	**(n = 8)**		**(n = 8)**	**(n = 8)**		**(n = 8)**	**(n = 8)**	
D1-2	55.3	0.4^4^	**99.3**	67.2	0.7^4^	**99.0**	62.0	0.0^4^	**100.0**	74.3	0.0^4^	**100.0**	67.4	0.3^4^	**99.5**
D8-9	79.4	0.0^4^	**100**	55.4	0.0^4^	**100.0**	79.4	0.0^4^	**100.0**	86.9	0.0^4^	**100.0**	59.9	0.0^4^	**100.0**
D15-16	84.9	0.0^4^	**100**	64.9	0.0^4^	**100.0**	78.9	0.0^4^	**100.0**	85.9	0.0^4^	**100.0**	63.2	0.1^4^	**99.9**
D22-23	86.8	0.2^4^	**99.8**	68.0	0.0^4^	**100.0**	87.2	0.0^4^	**100.0**	97.9	0.0^4^	**100.0**	73.4	0.0^4^	**100.0**
D29-30	89.3	1.4^4^	**98.4**	62.0	0.0^4^	**100.0**	82.0	0.0^4^	**100.0**	89.8	0.0^4^	**100.0**	67.1	0.3^4^	**99.6**

^1^In study 1, fleas were counted and removed 24 h after treatment or infestation.

^2^In studies 2, 3,4 and 5, fleas were counted and removed 48 h after treatment or infestation.

^3^Geometric mean flea count.

^4^Treated group differed statistically significantly (p < 0.005) from the untreated Control group.

Dogs administered a single topical treatment with a combination of fipronil and permethrin and submitted or not to water exposure (either shampooed once during the month or water immersed two or three times) had significantly (p < 0.005) lower flea counts than untreated controls 24 h or 48 h post-treatment or post-infestation.

In study 1, dogs were not subjected to any water exposure. Flea counts performed 24 h post treatment/infestation showed an efficacy between 98.4 and 100% for the entire month.

In studies 2 and 3, dogs were subjected to 2 or 3 water immersions on D10 and D24 (and D17 for study 3) and efficacy evaluated 48 h post treatment or infestation was constantly > 99%.

In studies 4 and 5, dogs underwent one shampooing on D17. The flea efficacy evaluated 48 h post infestation or treatment was above 99.6% for the entire month. There were no adverse events associated with fipronil and permethrin treatments in any of the studies.

## Discussion

In five different studies, a single application of fipronil and permethrin provided very high efficacy levels ranging from 98.4% to 100% control of adult cat fleas on dogs for one month after treatment. In two of the studies (studies 3 and 4) efficacy was constantly maintained at 100% during the full month on dogs that underwent either three water immersions or one shampooing during the study. Veterinarians and pet owners should expect that when correctly applied, this new combination of fipronil and permethrin spot-on will control the existing flea burden on a dog even under conditions of natural water exposure or shampooing once a month.

Several strains of fleas were used in different laboratories from different geographical areas in order to reflect the diversity of *Ctenocephalides felis* populations. No significant differences were observed between the studies either for infestation rates or for efficacy results. Slight variations were observed in the flea counts from control groups but these variations were related to natural variation observable in such experiments.

Fipronil and permethrin translocate on the skin within 24 h and accumulate in skin lipids. Their lipophilic properties and dose were studied to provide a month of protection against fleas and ticks under natural conditions including water exposure and shampooing [6]. Water immersion is an important factor to be considered when assessing the efficacy of an ectoparasiticide in the field. Indeed, animals exposed to parasitic pressure usually have an outdoor lifestyle and are therefore often subjected to natural conditions including rain, swimming and bathing. In the different studies presented here, the first exposure to water occurred 10 days after treatment and the effect of earlier water or shampoo exposures was not evaluated. It has been demonstrated by radio labelling that fipronil concentrates in the superficial skin layers including stratum Malpighii and stratum corneum [11] which provide an impermeable outer layer to the organism. This suggests that once absorbed into the skin, the molecules are protected from water challenge. Therefore, similar results would be expected when water exposure occurs between 24 h and 10 days post-treatment. As no data are available yet on the speed of skin absorption of the compounds, the effect of water exposure occurring within the first hours post-treatment remains unknown and should be avoided.

This study underscores the need to choose the appropriate antiparasitic treatment adapted to each dog’s lifestyle and environment [7].

As the risk for co-infestation by several species or group of parasites is a concern for a vast majority of dogs, there is a need for broad spectrum protection [1]. The efficacy of the new combination of fipronil and permethrin against the dog flea, *C. canis,* was also demonstrated in another study [12]. In addition, the efficacy of the product as a long lasting repellent against a range of hematophageous diptera including sandflies (*Phlebotomus perniciosus*), mosquitoes (*Aedes* spp., *Culex pipiens*) and stable flies (*Stomoxys calcitrans*) [13-15] has been demonstrated, as well as a long lasting efficacy against *Ixodes ricinus, Dermacentor reticulatus*, and *Rhipicephalus sanguineus*, the main tick species infesting dogs in Europe [16,17].

## Conclusions

In conclusion, this new combination adds the repellent properties of permethrin to the strong insecticidal and acaricidal efficacy of fipronil and offers broad spectrum protection against the major ectoparasites of dogs, including a high level of efficacy against *C. felis* fleas as demonstrated in the studies presented here.
